# Optimal use of plasma and urine BK viral loads for screening and predicting BK nephropathy

**DOI:** 10.1186/s12879-016-1652-6

**Published:** 2016-07-22

**Authors:** Peter Boan, Christopher Hewison, Ramyasuda Swaminathan, Ashley Irish, Kevin Warr, Rajalingam Sinniah, Todd M. Pryce, James Flexman

**Affiliations:** Departments of Microbiology and Infectious Diseases, PathWest Laboratory Medicine WA and Fiona Stanley Hospital, Level 1, Path Block, Fiona Stanley Hospital, 102-118 Murdoch Dve, Murdoch, Perth, 6150 Western Australia Australia; Department of Microbiology, PathWest Laboratory Medicine WA, Level 1, Path Block, Fiona Stanley Hospital, 102-118 Murdoch Dve, Murdoch, Perth, 6150 Western Australia Australia; Renal Transplant Unit, Fiona Stanley Hospital, 102-118 Murdoch Dve, Murdoch, Perth, 6150 Western Australia Australia; Department of Histopathology, Royal Perth Hospital, GPO Box X2214, Perth, 6000 Western Australia Australia

**Keywords:** BK virus nephropathy, BK viraemia, BK viruria, Polyoma virus, Renal transplantation

## Abstract

**Background:**

BK virus is a polyoma virus causing renal allograft nephropathy. Reduction of immunosuppression with the early recognition of significant BK viral loads in urine and plasma can effectively prevent BKV associated nephropathy (BKVN), however the optimal compartment and frequency of BK viral load measurement post renal transplantation are undetermined. Our purpose was to examine time to detection and viral loads in urine compared to plasma, and establish viral load cut-offs associated with histological BKVN.

**Methods:**

We performed a retrospective analysis of the BKV screening frequency and compartment(s) of 277 adult renal transplant recipients (RTR).

**Results:**

BKVN was histologically diagnosed in 17 (6.1 %) RTR. In cases where both urine and plasma were tested fortnightly for 6 months (*n* = 53), BKV was detected in the urine 29 days earlier than plasma. Fortnightly (*n* = 72) versus 3-monthly (*n* = 78) testing demonstrated that BKV was detected in the urine significantly earlier (median 63 versus 97 days, p = 0.001) and at a lower level (median 3.27 versus 6.71 log_10_ c/mL, *p* < 0.001) with more frequent testing, but this difference was not evident in plasma first detection (80 versus 95 days, *p* = 0.536) or first positive viral load (3.18 versus 3.30 log_10_ c/mL, *p* = 0.603). The optimum cut-off BK viral load for histological diagnosis of BKVN was 4.10 log_10_ c/mL for the first positive urine, 3.79 log_10_ c/mL for the first positive plasma, 9.24 log_10_ c/mL for the peak urine, and 4.53 log_10_ c/mL for the peak plasma.

**Conclusions:**

Frequent urinary BK viral load screening for the prevention of BKVN is suggested due to its high sensitivity and earlier detection.

## Background

BK virus (BKV) is a ubiquitous polyoma virus that causes a clinically important pattern of renal allograft injury in approximately 5 % of renal transplant recipients (RTR). Although viral replication can occur in up to 60 % of recipients [1], in its most severe form, invasive disease leads to tubulointerstitial nephritis and allograft dysfunction with a significant risk of allograft failure in 15–50 % of affected individuals. Recognised risk factors for BK virus nephropathy (BKVN) include the intensity of immunosuppression, treatment of acute rejection, male gender, ureteral trauma, and diabetes mellitus [2]. Strategies involving early screening and pre-emptive management (mainly reduction in immunosuppression) can prevent long term graft dysfunction due to invasive disease [2]. Once BKVN is established, treatment options apart from reduction in immunosuppression are unproven and largely untested by clinical trials. Measures for the early identification of risk in order to prevent progression of viral replication and progressive infection are therefore preferred.

Screening for BK virus can be performed by examination for urinary decoy cells, Haufen on electron microscopy of the urine, or more commonly by urine or plasma detection of virus by molecular methods. Recommendations on the suggested frequency of screening are largely opinion based but in general suggest 1–3 monthly in the first 6 months post transplantation followed by 3 monthly testing until 1 to 5 years post transplantation [2–4]. Definitive evidence of BKVN requires histological evidence of viral injury and exclusion of other causes of graft dysfunction by renal biopsy. Typically, extensive tubulointerstitial nephritis with positive immunohistochemical staining of the SV40 large T antigen is noted, however disease can be focal and missed by renal biopsy if sample size is inadequate [2]. Some studies suggest that BKVN can be presumptively diagnosed when the plasma viral load exceeds 10^4^ copies/mL [2, 4]. However in the absence of a quantitative international standard for BK virus assays, such cut-off values for individual assays need to be validated at an institutional level.

In Western Australia, the two renal transplant services independently introduced different screening strategies such that the renal transplant centre at Royal Perth Hospital performed fortnightly BKV screening of both urine and plasma for the first 6 months post transplantation, whilst Sir Charles Gairdner Hospital tested 3-monthly urine and plasma for the first 6 months. The introduction and adherence to these strategies varied over time within both centres. This “quasi-randomisation” by hospital allows unique comparison of the performance of different compartments and frequencies utilised in screening for BKVN.

## Methods

We performed a retrospective cohort study of the 277 consecutive adult renal transplants performed over the 5 year period between January 2008 and December 2012 at Royal Perth and Sir Charles Gairdner Hospitals who had at least 12 months clinical follow up. We extracted laboratory data from the electronic pathology results and patient clinical records and recorded the histological diagnoses and scores assigned to renal biopsies (performed for protocol or investigation of renal dysfunction), specifically relating to the coding of acute rejection and BKVN. We recorded all BKV plasma and urine viral load results and screening frequency then determined the timing and level of the first positive and the peak BK viral load for urine and plasma. Data were evaluated to 31 December 2013.

In Western Australia patients receive Basiliximab induction and routine triple immunosuppression with Tacrolimus, Mycophenolate sodium or mofetil and oral Prednisolone. Target levels are usually 5–10 mcg/L for Tacrolimus for the first 3 months and Prednisolone is tapered to 5–10 mg daily by 3 months.

All renal histology was performed by histopathologists trained in renal histology and reported for the purposes of clinical management only. Histology diagnosed as acute rejection (borderline, grade I-III), acute humoral rejection or BKVN were coded dichotomously as biopsy-proven acute rejection (BPAR) rejection (yes/no) and BKVN (yes/no). All BKVN cases demonstrated positive immunostaining against SV40. Rejection episodes diagnosed by biopsy were managed with an increment in steroids as intravenous pulses, and for unresponsive cases additional treatment with anti-T cell therapies were commenced.

The four value MDRD study equation for creatinine values standardised to creatinine reference materials was used to calculate eGFR in mL/min per 1.73 m^2^ = 175 × SCr^-1.154^× Age^0.203^ × (0.742 if female) × (1.21 if black). We did not apply the correction factor for “black” race to Aboriginal and Torres Strait Islanders as there is insufficient evidence it applies to this specific indigenous group.

### Molecular methods

Samples were EDTA plasma or sterile urine. Extraction, polymerase chain reaction (PCR) and detection was performed as previously described with some modifications [5]. Briefly, 200 μL of sample was extracted with the MagNaPure LC instrument using the Total Nucleic Acid Isolation Kit. A 50 μL volume of elution buffer was used. A total of 5 μL of eluted nucleic acid was added to 15 μL of PCR mix comprising 2 μL FastStart 10X Reaction Mix Hybridisation probes, 2 μL MgCl_2_ at 25 μmol/L, 1 μL each of forward and reverse primer at 20 μmol/L, 0.2 μL BKV probe at 20 μmol/L and 8.8 μL of water. PCR Amplification of the BK virus capsid protein-1 (VP-1) gene was performed using the LightCycler 1.5 instrument with 45 cycles amplification at 95°C 10 s, 60°C 20 s, 72°C 15 s. Oligonucleotide sequences used for the BKV forward primer were 5’ GCA GCT CCC AAA AAG CCA AA 3’, for the BK reverse primer were 5’ CTG GGT TTA GGA AGC ATT CTA 3’, and for the BKV probe were 5’ 6FAM--ACC CGT GCA AGT GCC AAA ACT AC--BHQ1 3’. An entire genome of Dunlop strain BKV in a pBR322 plasmid served as a quantitative standard of 1 × 10^6^ copies/reaction with a crossing point of 22 cycles. Positive and negative controls were utilised for each PCR run. The lower and upper limits of quantification are 2.7 log_10_ and 9.87 log_10_ copies/mL.

### Statistical methods

Categorical variables were compared with Fisher’s exact test, and continuous variables by Mann Whitney U test (of log_10_ results in the case of viral load measurements). Urine and plasma viral load association were determined by simple linear regression and determination of Pearson’s correlation coefficient after log-transformation. Receiver operator characteristic analysis was performed according to DeLong [6]. MedCalc version 12.4.0.0 was used for statistical analysis. Statistical significance was defined by p-value <0.05 with two-tailed tests.

### Ethical considerations

The study was approved by the Human Research Ethics Committee of Royal Perth Hospital and was conducted according to the principles of the National Statement on Ethical Conduct in Human Research [7].

## Results

### Patient characteristics

Excluded were 28 renal transplants who failed to reach 12 months follow up due to death (*n* = 6), graft loss (*n* = 8), loss to follow up (*n* = 14). 23 transplants had at least one renal biopsy, none with BKVN. Baseline data of the included 277 renal transplants with and without BKVN with at least 12 months follow up are presented in Table 1. Death occurred in 0 versus 20 (*p* = 0.621), graft loss 1 versus 8 (*p* = 0.439), and loss to follow up 0 versus 2 (*p* = 1.000) BKV positive versus BKV negative transplants respectively. BK viral load screening frequency in the first 6 months after renal transplantation was fortnightly (*n* = 72), monthly (*n* = 22), 3 monthly (*n* = 78), 6 monthly (*n* = 27) and nil (*n* = 78).

**Table 1:** Characteristics of renal transplants with and without BK nephropathy

Characteristic	BK nephropathy (*n* = 17)	No BK nephropathy (*n* = 260)	*p*-value
Age (years)	58.3 (47.8–61.6)	51.2 (41.6–58.1)	0.113
Male	12 (70.6)	157 (60.4)	0.454
ATSI^a^	0 (0)	31 (11.9)	0.232
Follow up (years)	4.1 (2.1–4.9)	3.4 (2.4–4.8)	0.966
Acute Rejection	7 (41.1)	56 (21.5)	0.074
eGFR at 12 months (mLs/min)	35.6 (22.3–52.2)	48.3 (38.6–59.8)	0.014

Results are median (percentage or interquartile range)

^a^ATSI, aboriginal or Torres Strait Islander

### BK nephropathy cases

BKVN was confirmed histologically in 17 (6.1 %) transplants. The median time to a histological diagnosis of BKVN was 233 days: in 4 patients virologically screened fortnightly this was 55, 67, 136, 283 days compared with eight patients screened every 3 months at 89, 99, 103, 170, 194, 233, 271, 324 days (*p* = 0.368). The time to BKVN was 469, 920, 1113 days in the three cases with no screening in the 6 months post transplantation, 806 days for one case screened 6 monthly, and 666 days for one case screened monthly. Patients with BKVN had a non-significant trend towards higher reported BPAR and significantly reduced eGFR at 12 months (Table 1). There were no episodes of BPAR following the diagnosis of BK nephropathy.

### Urine compared with plasma viral load screening

**Fig. 1:**
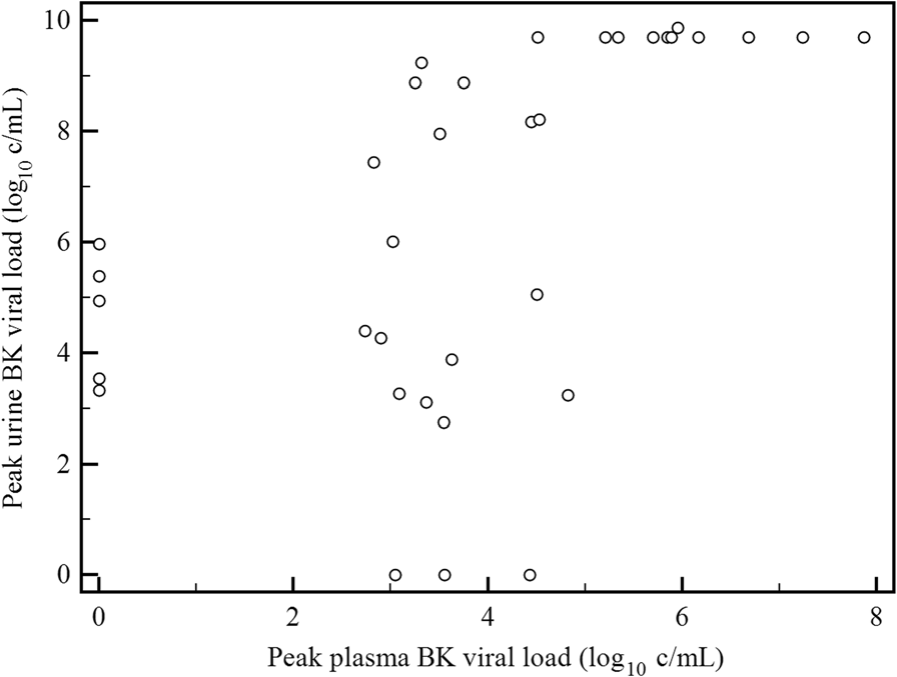
Correlation of peak plasma and urine BK viral load (log_10_ copies/mL). Legend: Correlation coefficient 0.53, 0.24–0.74 95 % CI, *p* = 0.001, y = 3.2119 + 0.8353×

In the 53 cases where urine and plasma were screened fortnightly for the first 6 months post transplantation, BKV was detected in the urine of 32 cases and in the plasma of 30 cases. In 27 cases BKV was detected in both urine and plasma. In five cases BKV was only detected in the urine with peak viral loads of 3.3, 3.5, 5.0, 5.4 and 6.0 log_10_ copies/mL (c/mL). In three cases BKV was only detected in plasma, with peak viral loads of 3.0, 3.6, and 4.4 log_10_ c/mL. Median time to the first positive urine BK viral load was shorter compared to the first positive plasma BK viral load (61 [25–104 interquartile range {IQR}] versus 90 [54–148 IQR] days, *p* = 0.066). The time between the first positive viral load and the peak viral load did not differ within the urine and plasma compartments (38 [3–113 IQR] versus 41 [0–153 IQR] days, *p* = 0.754). Urinary viral loads commonly reach the upper quantifiable limit of the test (log_10_ 9.87 c/mL) which limits the precision of this calculation. However despite this limitation there was a significant correlation between the peak urine and plasma viral load of the 35 cases where both compartments were screened fortnightly and BKV was detected in at least one compartment (correlation coefficient 0.53, *p* = 0.001, 0.24–0.74 95 % CI, y = 3.2119 + 0.8353×; Fig. 1).

### Comparison of screening frequencies

**Fig. 2:**
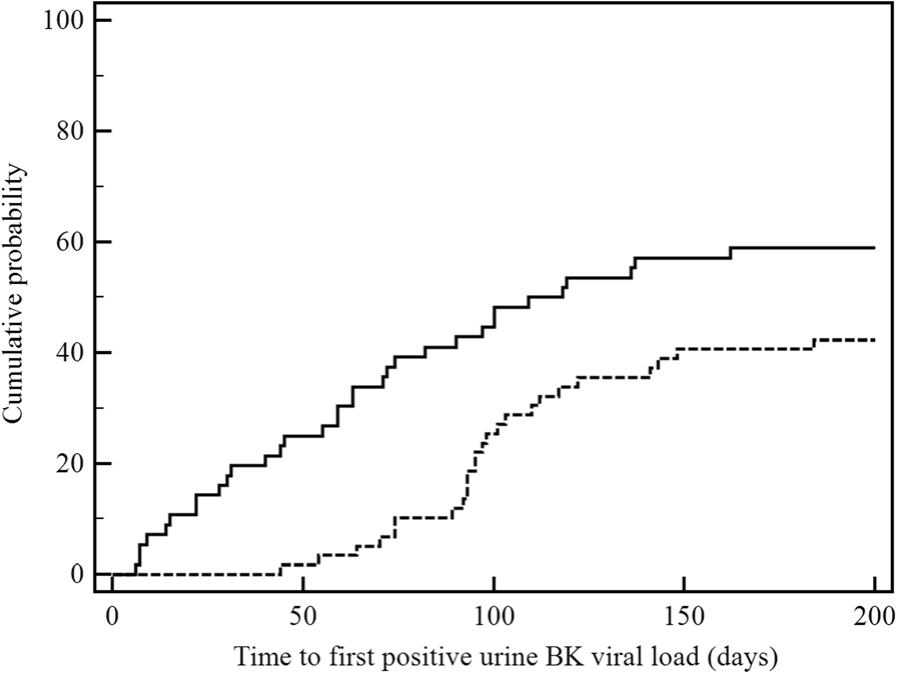
Time to first positive BK urine viral load, fortnightly vs 3-monthly testing. Legend: Kaplan-Meier curve of time to first positive BK urine viral load in the first 200 days post transplantation for patients tested fortnightly (*solid line*) and 3-monthly (*dashed line*)

There was no significant difference in the prevalence of BKVN or eGFR at 12 months between fortnightly versus the 3-monthly screening strategies (Table 2). Urine viral loads were detected earlier (Fig. 2) and at lower levels in the fortnightly group, while first positive plasma viral loads were similar between the two groups (Table 2). The incidence of BKVN was 3.8 % in the 78 patients where no virological screening was performed although the surveillance by protocol or for cause renal biopsy was similarly high between those cases where no screening versus any screening was performed (87.2 % versus 84.9 %, *p* = 0.707).

**Table 2:** Comparison of fortnightly and 3-monthly BK viral load screening strategies

Characteristic	Fortnightly (*n* = 72)	3-monthly (*n* = 78)	*p*-value
First positive urine viral load	3.27 (3.03–4.25)	6.71 (4.90–8.23)	<0.001
First positive plasma viral load	3.18 (2.88–3.78)	3.30 (2.89–4.19)	0.603
Peak urinary viral load	7.96 (4.54–9.70)	7.88 (5.76–9.52)	0.911
Peak plasma viral load	3.85 (3.20–5.28)	4.23 (3.26–5.11)	0.965
Time to first positive urine viral load	63 (28–100)	97 (90–131)	0.001
Time to first positive plasma viral load	80 (52–155)	95 (73–120)	0.536
eGFR at 12 months	47.0 (34.1–57.9)	44.9 (35.8–60.0)	0.870
BK nephropathy	4 (5.55)	8 (10.26)	0.372

Results are median (interquartile range), except BK nephropathy which is number (%). Viral loads are log_10_ copies/mL. Viral load data represent those cases with a detectable viral load where the compartment was used for screening

### Urine and plasma viral loads for predicting BK nephropathy

Utilising only cases with a detectable viral load undergoing 3 monthly or more frequent screening of the relevant compartment, we found the first and peak urinary and plasma viral loads were significantly higher in those cases with BKVN (Table 3).

**Table 3:** First and peak urine and plasma viral loads with and without BK nephropathy

Viral load	BK nephropathy	No BK nephropathy	*p*-value
First positive urine	7.79 (5.05–9.70)	3.54 (3.10–6.70)	0.001
Peak urine	9.70 (9.70–9.70)	6.72 (4.37–9.00)	<0.001
First positive plasma	4.81 (3.73–5.40)	3.05 (2.86–3.68)	<0.001
Peak plasma	5.73 (5.25–6.75)	3.63 (3.01–4.53)	<0.001

Viral loads are median (interquartile range) log_10_ copies/mL. Data represents those transplants with a detectable viral load undergoing 3-monthly or more frequent testing of the compartment in the first 6 months post transplantation. BK nephropathy: urine testing (*n* = 11), plasma testing (*n* = 13). No BK nephropathy: urine testing (*n* = 115), plasma testing (*n* = 153)

**Fig. 3:**
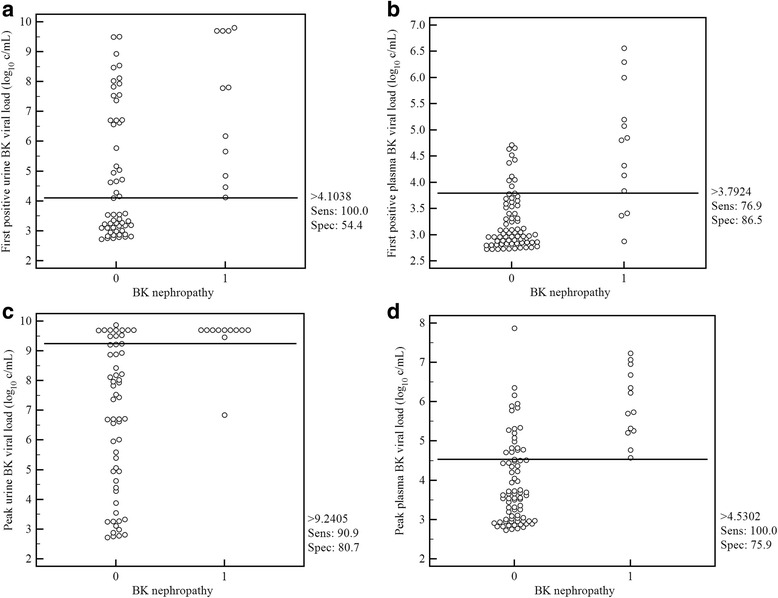
BK viral load relationship to BK nephropathy. Legend: Dot diagrams with Receiver Operator Characteristic analysis of those without (0) and with (1) BK nephropathy of: **a** first positive urine BK viral load **b** first positive plasma BK viral load **c** peak urine BK viral load **d** peak plasma BK viral load. Viral loads are log_10_ copies/mL. Horizontal lines designate optimum cut-offs. Sens = sensitivity, Spec = specificity

Receiver operator characteristic analysis suggested optimal cut-offs for BKVN of 4.10 log_10_ c/mL for first positive urine, 3.79 log_10_ c/mL for first positive plasma, 9.24 log_10_ c/mL for peak positive urine, 4.53 log_10_ c/mL for peak positive plasma (Table 4, Fig. 3a, b, c, d).

**Table 4:** Receiver Operator Characteristic analysis of optimal viral load cut-offs for detecting BK nephropathy

Viral load	Cutoff^a^	Sensitivity	Specificity	AUC^b^
First positive urine	4.10	100	54	0.80
Peak urine	9.24	91	81	0.88
First positive plasma	3.79	77	81	0.85
Peak plasma	4.53	100	76	0.92

^a^Viral loads in log_10_ copies/mL. ^b^AUC = area under the curve

## Discussion

We examined and compared the frequency and compartment of BK viral load testing in relation to BKVN diagnosed by renal histopathology and defined potential receiver operator characteristic derived cut-offs for diagnostic accuracy. In our subset of 53 cases where fortnightly testing of both urine and plasma were performed, BKV was detected 29 days sooner in urine than plasma. Therefore monitoring of urine allows the earliest identification of viral replication, in agreement with other studies [8, 9]. Subgroup analysis of 14 cases by Funk et al. found BKV in the plasma became detectable 50 days after the urine compartment, however the frequency of testing and time from transplant were not recorded.^8^ Babel et al. recorded viremia 6 weeks after viruria from 233 patients tested monthly for BKV in blood and urine [9].

It was anticipated that more frequent screening (fortnightly versus 3-monthly) would detect BKV in the urine earlier and at a lower viral load as shown in our study, however we demonstrate that there was no statistical difference in the time to first detection or the initial viral load for the plasma compartment between the two screening frequencies. We reason that this discrepancy relates to BKV appearing in the plasma much later than in urine such that less advantage is found by more frequent plasma screening early post transplantation. We could not detect a difference in BKVN prevalence between these two screening frequencies although this may be due to insufficient sample size. We cannot explain the low prevalence of BKVN demonstrated for those transplants in whom no virological screening was performed. Although renal biopsy was performed for a similar percentage of these transplants, it is possible there was less active case-finding associated with unmeasured variables in this group. We do not advocate cessation of BK viral load screening on account of this finding of uncertain significance.

We confirmed that our in-house BKV quantitative assay is able to predict the risk of histologically proven BKVN and show that the ROC analysis identifies an optimal viral load cut-off for BKVN of 4.10 log_10_ c/mL for the first positive urine, 3.79 log_10_ c/mL for first positive plasma, 9.24 log_10_ c/mL for peak urine and 4.53 log_10_ c/mL for peak plasma. Kudose et al. obtained similar optimal BK viral load cutoffs of 7.2 and 3.7 log_10_ copies/mL in urine and plasma respectively for the detection of BKVN in concurrent biopsy [10], another study identifying plasma BK viral load of 4.1 log_10_ copies/mL as the best discriminator of BKVN [11]. Therefore viral load cut-offs proposed in the literature of 7 log_10_ c/mL in urine and 4 log_10_ c/mL in plasma are similar to our results [2, 3, 12, 13], after accounting for our difference between initial and peak cut-offs.

However since the object of screening is to prevent the development of BKVN, based on our data, we deduce that the urine must be utilised if regular screening is performed early post transplantation to provide an advantage in earlier detection of BKV at a low viral load. There is no advantage in regular screening of plasma because there was no significant difference found in the time to first detection or initial viral load between fortnightly and 3-monthly testing. Additionally in contrast to the urine compartment, there is little difference in initial and peak ROC derived plasma viral load cut-offs for BKVN, suggesting the moment BKV is detected in the plasma the patient is at imminent risk for BKVN. If only plasma is screened, there is limited lead time for intervention to prevent BKVN. Adding to these concerns of relying on testing of the plasma compartment to screen or presumptively diagnose BKVN is a single study showing 11 of 31 patients with BKVN had plasma BK viral loads consistently <4 log_10_ copies/mL [14].

Accordingly, our adopted practice is regular urine BK viral load testing as the primary screening strategy due to its high sensitivity and earlier detection than the plasma compartment, with restriction of plasma testing to patients with a urine viral load exceeding 7 log_10_ c/mL. We suggest that utilising a urine first strategy provides an early warning to clinicians to take measures to modify the risk of progressive BKV replication. We confirm that a plasma BK viral load greater than 4 log_10_ c/mL is predictive of a risk of BK nephropathy and should prompt diagnostic biopsy and significant modification of immunosuppression or additional therapy according to the clinical situation. This study validates the approach previously proposed by Hirsch et al. [2].

The major limitation of the study relates to the reliance on histopathology for the diagnosis because histopathological changes occur late in the disease and disease can be missed by biopsy due to focal involvement. Biopsies were performed at the discretion of the treating physician rather than as part of a protocol and the decision to biopsy the patient may have been influenced by the BK viral loads leading to ascertainment bias. Due to logistical constraints, we lack information about risk factors for the development of BKVN and the management of presumptive or confirmed BKVN, however this was not the aim of this study. The transplants screened 3-monthly and fortnightly were largely from two different transplant centres, and BKV management was not protocolised across the centres. Bias according to centre could have potentially contributed to the outcomes of the two groups.

As the presence of BKV antigen-specific T-cell responses are associated with control of BKV replication [15–18], and there are scant reports of the association of BKV genotype with BKVN [19, 20], with further study it would be interesting to evaluate BKV specific T-cell immunity and BKV genotype for differences in cases with low or undetectable BK viral loads, high BK viral loads without BKVN, and those cases with BKVN.

## Conclusions

In this retrospective observational study, we have not been able to demonstrate that better clinical outcomes are associated with more frequent BK viral load testing or testing of a particular compartment. However we have shown if frequent BK viral load testing is employed early post transplantation, the advantage of earlier detection is found for urine but not plasma testing. Additionally, the initial detection of BKV in the plasma occurs at a level already predicting a risk of BKV nephropathy. For these reasons we suggest urine rather than plasma as the optimal compartment for BK viral load screening.

## Abbreviations

ATSI, aboriginal or Torres Strait Islander; BKV, BK virus; BKVN, BK virus nephropathy; c/mL, copies per millilitre; CI, confidence interval; IQR, interquartile range; PCR, polymerase chain reaction
